# The Ability of First Aid Providers to Recognize Anaphylaxis: A Scoping Review

**DOI:** 10.7759/cureus.41547

**Published:** 2023-07-08

**Authors:** Daniel Meyran, Pascal Cassan, Michael Nemeth, Eunice Singletary, James Raitt, Therese Djarv, Jestin N Carlson

**Affiliations:** 1 Healthcare, French Red Cross, Paris, FRA; 2 Prehospital Emergency Care, Bataillon De Marins Pompiers De Marseille, Marseille, FRA; 3 Prehospital Emergency Care, International Federation of Red Cross and Red Crescent Societies (IFRC) Global Reference First Aid Reference Center, French Red Cross, Paris, FRA; 4 Prehospital Care, International Liaison Committee on Resuscitation, Toronto, CAN; 5 Emergency Medicine, University of Virginia, Charlottesville, USA; 6 Emergency Medicine, Thames Valley Air Ambulance, Oxford, GBR; 7 Emergency Medicine, Karolinska Institute, Stockholm, SWE; 8 Emergency Medicine, Saint Vincent Hospital, Erie, USA

**Keywords:** lay provider, diagnosis, first aid, prehospital, recognition, anaphylaxis

## Abstract

Early recognition of anaphylaxis is critical to early treatment and often occurs in the first aid setting. However, the ability of first aid providers to recognize anaphylaxis is unknown. We sought to examine the evidence regarding first aid providers’ ability to recognize anaphylaxis.

Our scoping review was performed as part of the International Liaison Committee on Resuscitation (ILCOR) continuous evidence evaluation processes to update the 2020 ILCOR Consensus on Science with Treatment Recommendations. We searched Medline, Embase, Cochrane, and the gray literature from 2010 to September 2022. The population included adults and children experiencing anaphylaxis with a description of any specific symptom to a first aid provider. Recognition of anaphylaxis was the primary outcome. Two investigators (DM and PC) reviewed abstracts and extracted and assessed the data. Discrepancies between the reviewers were resolved by discussion and consensus with the ILCOR First Aid Task Force.

Out of 957 hits, 17 studies met inclusion criteria: one review and meta-analysis, two experimental studies, and 14 observational studies. We did not identify any studies that directly addressed our PICOST (Population, Intervention, Control, Outcomes, Study Design, and Timeframe) as none were performed in the first aid setting. Articles included individuals who may be first aid providers as patients and parents (n=5), teachers, students or school staff (n=8), caregivers and patients (n= 2) or nannies (n=1). All included studies were conducted in high-income countries. Our scoping review found that signs and symptoms of anaphylaxis were not specific and did not allow for easy identification by the first aid provider. Studies focused on education (n=10) and protocols (n=2) and found that both could have a positive impact on anaphylaxis recognition and management.

While we did not identify any clinical studies that directly addressed the ability of first aid providers to identify anaphylaxis, future studies examining education methods and action plans may help improve the identification of anaphylaxis by first aid providers.

## Introduction

Anaphylaxis is a serious, potentially life-threatening allergic reaction that can occur rapidly and unexpectedly and requires prompt medical treatment. Estimates suggest that up to 5.1% of the United States (US) population has experienced anaphylaxis [1,2]. Although there is no consensus on the incidence of anaphylaxis on a global scale, there is evidence of a global increase in the prevalence of anaphylaxis cases as well as hospitalizations [3]. Rates of hospitalizations for anaphylaxis in children have increased in many Western countries [4]. This may be due in part to improved recognition of common signs and symptoms [5], which can be variable depending on the cause of the anaphylaxis and the age of the patients [6].

Allergic reactions can advance to anaphylaxis within minutes. Therefore, it is essential for first aid providers to recognize the signs and symptoms of anaphylaxis for individuals to receive treatment in a timely manner. While epinephrine is the first line of treatment [7], a delay in treatment greater than 20 minutes is associated with an increase in fatal and near-fatal reactions [8].

Recognition of anaphylaxis can be difficult due to confounding definitions and diagnostic criteria which impact patient care practices. Furthermore, diagnosis can be challenging due to a wide constellation of symptoms that often mimic related allergic and non-allergic disorders [4]. Poor recognition and inadequate treatment by health professionals can lead to preventable errors and death. Studies show that both physicians [4,9] and prehospital providers have difficulty recognizing anaphylaxis [10,11]. Given the time-critical nature of epinephrine administration in anaphylaxis, it is important to understand the ability of first aid providers to recognize anaphylaxis.

Many signs and symptoms of anaphylaxis are typically described, helping first aid providers to recognize anaphylaxis. The most listed signs and symptoms reported for teaching to first aid providers from various international organizations are anxiety, breathing difficulties, including noisy breathing, wheezing or persistent cough, airway narrowing, swelling of the face and the tongue, difficulty talking and/or hoarse voice, abdominal pain, diarrhea, nausea and vomiting, hives, welts and body redness, signs of shock, including confusion or agitation, pallor and floppiness (young children), loss of consciousness, and cardiac arrest [12-15].

The most recent International Liaison Committee on Resuscitation (ILCOR) First Aid Consensus on Science with Treatment Recommendations (CoSTR) for this topic was published in 2010 and identified very low-certainty evidence from eight studies highlighting the limited ability of first aid providers to correctly identify anaphylaxis [16]. As part of the ILCOR First Aid Task Force (FATF), we performed a scoping review to identify studies evaluating or describing the ability of first aid providers to recognize anaphylaxis. The appropriate recognition of anaphylaxis by first aid providers is hoped to subsequently increase the use of epinephrine in this population.

## Materials and methods

Question and objectives

We sought to answer the population, interventions, comparators, outcomes, study design, timeframe (PICOST) question: Among adults and children experiencing anaphylaxis in the first aid setting, does the description of any specific signs or symptoms, compared with the absence of any specific description, increase the recognition of anaphylaxis by first aid providers? The objective of this scoping review was to examine the literature subsequent to the 2010 ILCOR CoSTR [16] and to establish whether there was new evidence to warrant a systematic review. This scoping review was performed as part of the ILCOR continuous evidence evaluation process, conducted by the ILCOR FATF Scoping Review team for the 2023 CoSTR.

Inclusion and exclusion criteria

Our population included adults and children experiencing anaphylaxis with a description of any specific symptom provided by the person, family member, or any other witness to a first aid provider and in a prehospital setting. We excluded all studies where data collection occurred in an emergency department or other healthcare facility by the healthcare provider.

Information sources and search strategy

**Figure 1:**
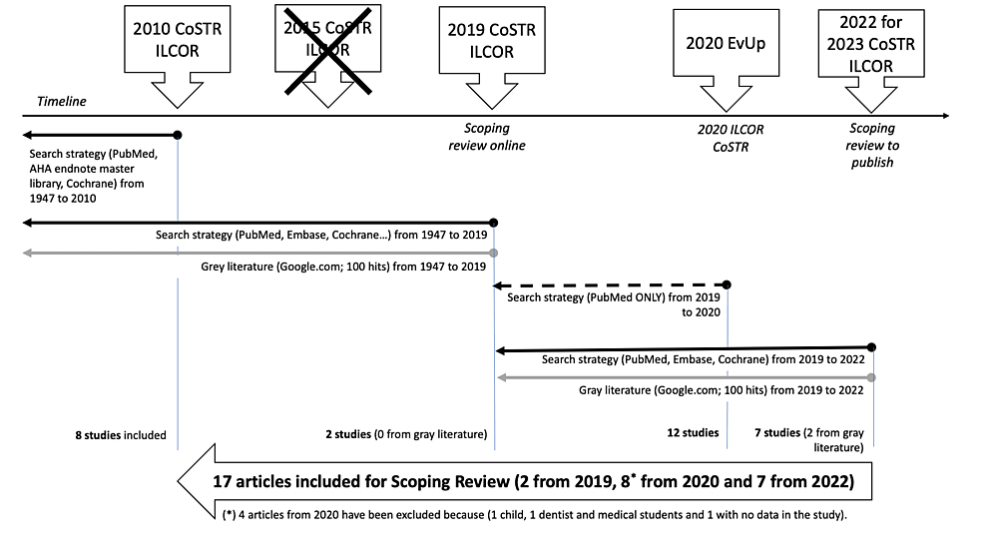
ILCOR FATF review strategy of the evidence from 2010 to 2022 Abbreviations: CoSTR, Consensus on Science with Treatment Recommendations; ILCOR, International Liaison Committee on Resuscitation; FATF, First Aid Task Force; EvUp, Evidence Update; AHA, American Heart Association.

The broad topic of anaphylaxis recognition by first aid providers was reviewed by ILCOR in 2010 [16]. As the initial review lacked identification of specific signs and symptoms that may abet the recognition of anaphylaxis, the ILCOR FATF created a revised search strategy in 2019 to review evidence from both published and gray literature (Figure 1). Two articles were included in a subsequent scoping review [17]. 

In 2020, the ILCOR FATF executed a time-limited search in PubMed for an evidence update. No new articles were found to directly answer the research question, but 12 studies were identified on educational interventions to improve the recognition of anaphylaxis. 

The current scoping review search strategy seeks new evidence in published and in the gray literature from 2019 to 2022 (Appendices). Articles identified using the current search strategy have been combined with studies identified in the previous searches for a comprehensive scoping review of all published and gray literature identified since 2010.

Published literature

The last published literature search using PubMed, Embase, and Cochrane was conducted on September 19, 2022 and updated on April 1, 2023. We included all human studies with no restriction on the language if there was an English abstract. Randomized controlled trials (RCTs) and non-randomized studies (non-RCTs, interrupted time series, controlled before-and-after studies, cohort studies) were eligible for inclusion. Unpublished studies (e.g., conference abstracts, trial protocols) were excluded unless subsequently retrieved in the gray literature search.

Gray literature

The last gray literature search of Google.com was conducted on September 30, 2022. We performed a structured search from November 2019 to September 2022. The first 100 hits from each search were reviewed to identify additional relevant material. 

Screening and selection of sources

For the published literature search, two independent reviewers (DM and PC) screened the title and abstract of each article. Then, the same reviewers performed a full-text review of potential articles to determine the final articles to be included. We manually reviewed references from all included studies. For the gray literature, one reviewer (DM) performed the initial search and identified potential sources. Two reviewers (DM and PC) then reviewed these sources to identify any additional key sources of information. Discrepancies between the reviewers were resolved by discussion with the ILCOR FATF. We present descriptive summaries of the final included studies.

## Results

**Figure 2:**
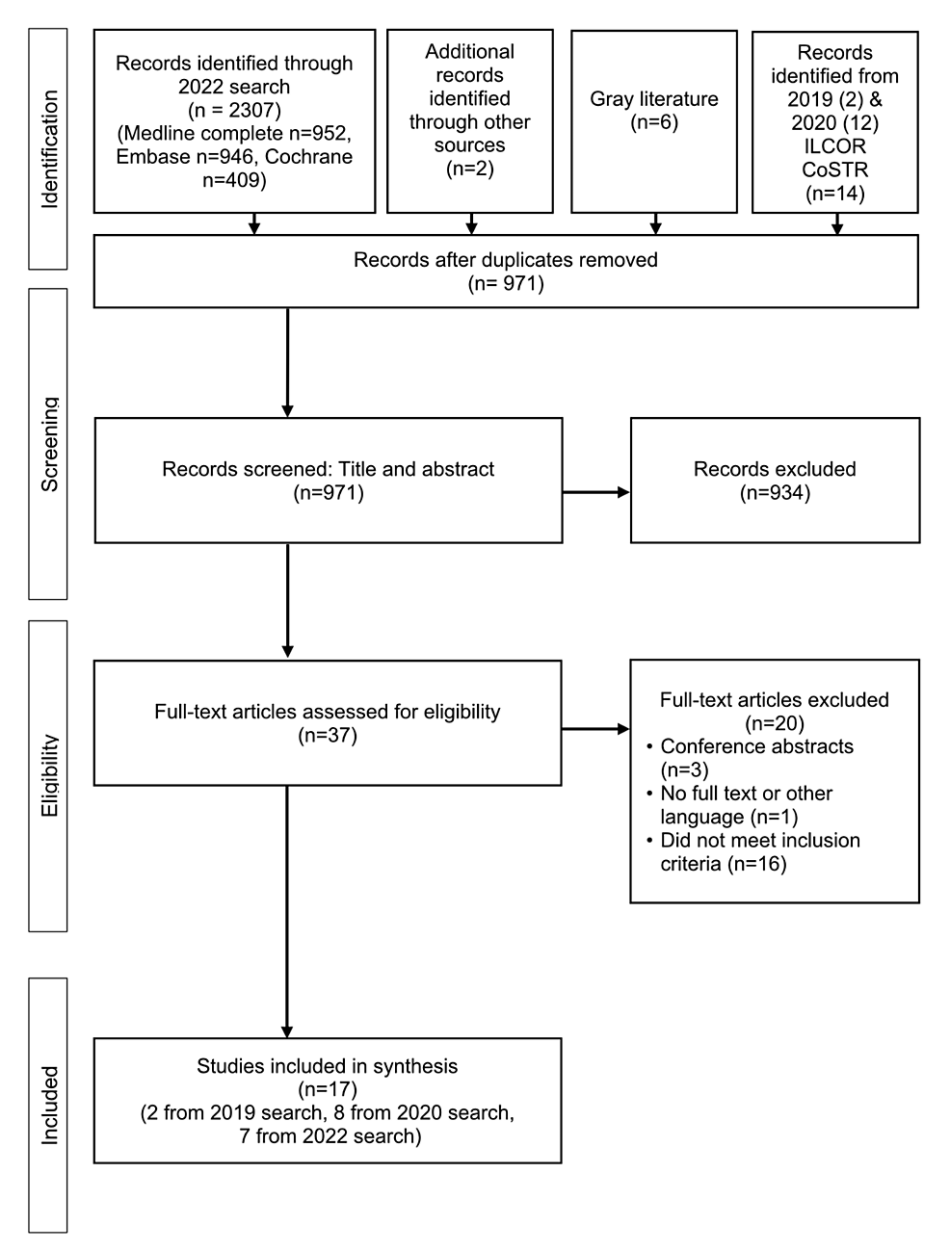
PRISMA flow diagram of included studies Abbreviations: PRISMA, Preferred reporting items for Systematic Reviews and Meta-Analyses, CoSTR, Consensus on Science with Treatment Recommendations; ILCOR, International Liaison Committee on Resuscitation.

For the literature search and study selection, the updated search strategy from 2019 to 2020 identified 949 unique titles/abstracts. We added two additional records identified in references from other included articles, six from gray literature, two selected studies from the 2019 rerun search strategy for the 2020 ILCOR FATF CoSTR [18], and 12 selected articles for the 2021 ILCOR evidence update. Based on titles and abstract screening, we excluded 934 studies. Of the 37 full-text articles reviewed, a further 20 were excluded, leaving a total of 17 studies, including two from the gray literature (Figure 2).

Study characteristics

Of the 17 articles included, we found one systematic review with meta-analysis [19], two experimental studies [20,21], and 14 observational studies [22-35]. The characteristics and findings of evidence are presented in Tables 1-3. All included studies were conducted in high-income countries. Nine studies concerned anaphylaxis secondary to food allergies [21,25,27-30,33-35], one study anaphylaxis secondary to food allergies and insect sting [26], and seven studies concerned anaphylaxis secondary to any type of allergy [19,20,22-24,31,32]. No study was performed in the first aid setting. Five studies evaluated patients and parents of affected patients [22,24,26,30,34], eight studies with teachers, school staff, or summer camp leaders [21,23,25,28,31-33,35], two studies with caregivers and their patients [20,27], and one study concerned nannies [29]. 

**Table 1:** Characteristics of guidelines or systematic reviews Abbreviation: CI, confidence interval

Author, Year, Location, Citation	Study design	Number of articles identified (n)	Population, type of allergy	Intervention	Key findings as presented in the article	Conclusions
Miles LM, 2021, Canada [19]	Review and meta-analysis.	(n=252) Epinephrine domain (44). Barrier domain (166). Cost-effectiveness (7). Program and domain strategy (35).	Community use of epinephrine autoinjectors in children and adults. All types of allergies.	Four domains: epinephrine use in the pre-hospital setting; barriers to epinephrine use in the pre-hospital setting; cost evaluation and cost-effectiveness of epinephrine use; programs and strategies to improve epinephrine use during anaphylaxis.	Epinephrine use in the prehospital setting was significantly higher for children compared with adults: 20.98% (95%CI: 16.38%, 26.46%) vs 7.17% (95%CI: 2.71%, 17.63%), respectively, P=0.0027). The pooled estimate of biphasic reactions among all anaphylaxis cases was 3.92% (95%CI: 2.88%, 5.32%). In reviewing programs and strategies, numerous studies have engineered effective methods to promote adequate and timely use of epinephrine.	Prehospital use of epinephrine in anaphylaxis remains suboptimal. Major barriers to the use of epinephrine were identified as low prescription rates of epinephrine autoinjectors and lack of stock of epinephrine in schools. The authors suggest increasing the use of epinephrine through stock supplies in schools and food courts, and using educational pamphlets in public areas to assist with recognition of anaphylaxis.

**Table 2:** Characteristics of experimental studies Abbreviations: RCT, randomized control trial; IG, intervention group; CG, control group; CI, confidence interval; FA, Food allergy;  OR, odds ratio.

Author, Year, Citation	Type of study	Location, study size (n)	Population, type of allergy	Intervention	Comparison	Outcomes	Key findings as presented in the article	Conclusions
Brockow, 2015 [20]	Multicenter RCT.	Germany (N=193).	Caregivers of affected children (95) and patients with previous episodes of anaphylaxis (98). All types of allergies.	Two-3h schooling modules of structure education program (IG).	Standard auto-injector training only (CG).	Knowledge of anaphylaxis. Emergency management competence. Secondary psychological parameters. All outcomes are assessed at baseline and 3 months after intervention.	In comparison with CG, the intervention led to significant improvement of knowledge for caregivers: IG, 3.2/13.2, improvement/baseline vs CG, 0.7/12.6; p<0.001; patients: IG, 3.9/10.8 vs 1.3/ 12.6; p <0.001. Emergency management competence was increased after intervention as compared to controls for: caregivers: IG, 8.6/11.2 vs CG, 1.2/ 10.8; p < 0.001; patients: 7.1/11.0 vs 1.1/11.1; p<0.001). The intervention showed a significant reduction of caregiver anxiety (-1.9/8.4 vs -0.7/7.5; p<0.05) but there were no significant changes in the depression scores.	Structured patient education programs may be beneficial in the management of anaphylaxis.
Canon N, 2019 [21]	Controlled before and after experimental study.	Houston, Texas, United States (n=375).	375 teachers from six private schools were assigned to an intervention (4 schools, n=302) and a control group (2 schools, n=73).	A 1-hour educational session on food allergies by one health care provider.	Pretest survey for the IG and CG. Post-test survey immediately after courses for the IG and 1 month after the pre-test for the CG (Survey: Chicago Food Allergy Research Survey).	Knowledge measure (linear mixed effect model). Attitudes (Likert scale). Beliefs (Likert scale)	Knowledge: The scores in the IG had 19,85% (95%CI, 16,62-22,53) points higher than the CG post-test (p<0.001). IG: the score is 19,78% (95%CI: 18,17-21,38) points higher in the post-test versus the pre-test (p<0.001). CG: no significant differences. Attitudes: In terms of an agreement that recognizing FA as a serious health problem, the post-test score was higher in the IG (16.3 times increased) compared with the control (p<0.001). Beliefs: Post-education, IG schools were 5 times more likely to recognize the difficulty of food avoidance in allergic patients compared to CG schools (OR=5.21; 95%CI, 73–15.70; p<0.003).	Short educational sessions improve the knowledge and attitudes of school staff and familiarize them with food allergies, early recognition of anaphylaxis, and the use of injectable epinephrine.

**Table 3:** Characteristics of the observational studies Abbreviations: UAP, Unlicensed Assistive Personnel; SD, standard deviation; IQR, interquartile range; EAI, Epinephrine Auto-Injector; FA, food allergy; FAM, food allergy management; AM, allergy management, CAP, Canadian action plan; GFI, Gunning Fog index; FRG, Fry Readability Graph; CIRF, Consumer Information Rating Form; EU, European Union; OFC, Oral Food Challenge.

Author, year, citation	Type of study	Location, study size (n)	Population, Type of allergy	Intervention	Comparison	Outcomes	Key findings as presented in the article	Conclusions
Litarowsky, 2004 [32]	Prospective before and after study.	United States of America (n=53).	Unlicensed assistive personnel in the high school setting. All types of anaphylaxis.	Training program to recognize and respond effectively to an anaphylactic emergency.	Test before and after the training program. No comparison group.	Knowledge and self-efficacy of personnel.	Knowledge: comparison of pre-test and post-test scores for knowledge showed significant improvement (p<0.001). The mean score of 5.28 (SD=1.769) at the pretest increased to 8.91 (SD=1.484) after the training intervention. Self-efficacy: Preintervention and postintervention perceived self-efficacy questionnaire scores showed significant improvement (p<0.001) upon comparison. The prequestionnaire mean of 20.06 (SD=7.315) increased to 35.69 (SD=4.213) after the training intervention.	A training program in the recognition of anaphylaxis and the use of EAI can improve the knowledge and self-efficacy of non-emergency response personnel.
Rodriguez Ferran L, 2020 [25]	Prospective before and after study.	Spain (n=53)	53 participants from three schools (85% teachers, 15% canteen staff). Food allergy.	Training session. 55 minutes of the theoretical part and 10 to 20 minutes of the practical part (use of EAI simulator).	Pre-and post-training questionnaires completed by participants before and after the training session. No comparison group.	Recognition of symptoms of allergic reaction. Recognition of anaphylaxis. Main medication of anaphylaxis. When to use EAI use. How to use an EAI. How to act after the use of EAI.	Frequency of correct answer (pre-training versus post-training questionnaire). Recognition of anaphylaxis: 40% vs 81% (p<0.001). Treatment of choice: 45,3% vs 79% (p<0.001). When to use EAI: 19% vs 100% (p<0.001). How to use EAI: 13% vs 100% (p<0.001).	Knowledge and confidence of staff who may have to respond to an anaphylactic reaction are significantly improved after simple training.
Polloni, 2020 [33]	Before and after study.	Italy (n=592).	Teachers and school caretakers. Food allergy.	2-hour course on first aid and anaphylaxis management with discussion.	Pre-and post-training questionnaires completed by participants before and after the training session. No control group.	Self-efficacy. AM: Recognition of anaphylaxis symptoms and administering proper good drug. FA management.	School personnel reported low self-efficacy in AM, especially in recognizing anaphylaxis symptoms and administering proper drugs. After training course, all scores improved, especially AM scores. Pre-Post difference total score for: AM and FAM score (median and IQR) = 6(3-9); AM score= 1(0.67-1.67); FAM score = 0.6(0,2-1). Significant difference in pre-post difference for recognition of anaphylaxis symptoms (p<0.05).	Specific multidisciplinary training courses may be effective to improve teachers' and school caretakers'’ self-efficacy in allergy management.
Dumeier 2018 [23]	Before and after study.	Germany (n=75).	Pre-school teachers. All types of allergies.	Education session (60 minutes with slides given before the session and an action plan given after the session).	Survey realized before, immediately after, and 4–12 weeks after the education session. No control group.	Assessment by the survey of experiences with allergies; knowledge of the disease allergy; attitudes towards and knowledge of anaphylactic emergency.	Results for the question “Which of the following descriptions represent possible symptoms of an anaphylactic emergency?” before, directly after and 4–12 weeks after. Anal incontinence and urinary incontinence: n= 17(23%)- 61(81%)*- 30(40%)**. Dip in blood pressure, dizziness: n=49(65%)- 68 (91%)*- 65(87%)**. Shortness of breath, wheezing: 56(75%)-60 (80%)-62(83%). Nausea, vomiting: n=44 (59%)- 67(89%)*- 60(80%)**. Swelling of skin and mucosa: n=73(97%)- 74(99%)- 73(97%). All 5 symptoms correctly related: n=7(9%)- 45(60%)*- 23(31%) (*p < 0.025, before vs. directly after education session; **p < 0.025, before vs. 4–12 weeks after education session).	A single education session substantially improved preschool teachers’ attitudes and knowledge of allergies, and anaphylactic emergencies and improves patient safety during drug administration.
Gonzalez-Mancebo E, 2019 [28]	Before and after study and anaphylaxis.	Spain (n=191).	Teachers (24%), cooks (13%), cafeteria monitors (51%), and summer camp leaders (12%). Food allergy.	Training course during a conference entitled ‘‘Management of Food Allergy in Children and Adolescents in School Centers”.	Questionnaire before and after the course to assess their self-efficacy in the management of food allergy and anaphylaxis.	Self-efficacy in management of food allergy and anaphylaxis.	The areas with the lowest confidence before receiving the course were recognition of symptoms and treatment of the reactions/anaphylaxis. The mean score for each of the eight concepts evaluated improved after the training course and this improvement was significant in the recognition of anaphylaxis symptoms. Comparison before and after the training was: Mean score (SD): 3.64 (1.14) – 4.56 (0.76) (p<0.05).	Training for school and canteen staff improves the recognition of anaphylaxis and the ability to manage food allergy and anaphylaxis.
Jiang, 2019 [30]	Before and after study.	United States of America(n=142).	Elementary (n=198), middle (n=156), and high school students from urban and private schools (n=203). Food allergy.	Peer-to-peer educational food allergy video (3 videos). Videos included top common food allergens, 18 symptoms of an allergic reaction, and EAI administration steps.	Pre- and post-tests assessing first aid knowledge.	Assess changes in first aid knowledge. Assess the efficacy of the videos as a learning tool.	Common food allergen: Elementary school: (n=195) 92.3% at pre-test vs. 96.4% at post-test, p<0.05; Middle school (n=133): 76.7% vs. 96.2%, p<0.001; High school (n=160): 88.1% vs. 96.9%, p<0.01. Common Anaphylaxis Symptoms: Elementary school: (n=192) 61.5% at pre-test vs. 85.9% at post-test, p<0.001; Middle school (n=147): 46.3% vs. 70.1%, p<0.001; High school (n=189): 66.1% vs. 85.2%, p<0.001). Epinephrine as appropriate medication for elementary school: (n=192) 66.1% at pre-test vs. 85.4% at post-test, p<0.001. Cross contact: Middle school (n=149): 79.2% vs. 96.0%, p<0.001; High school (n=196): 93.9% vs. 99.0%, p<0.01.	Peer educational videos are a useful tool in introducing first-aid concepts and improving first-aid knowledge, especially for the management of anaphylaxis.
Gallagher, 2019 [26]	Before and after study.	United States of America(n=22).	Adolescents at risk for anaphylaxis (n=22). Food and stinging insect allergy.	Use of a smartphone-based interactive teaching tool with decision support and EAI.	Before and after use of the smartphone application.	Decision support’s ability to improve allergic reaction management knowledge. Assess an EAI training module (participant’s ability to correctly demonstrate the use of an EAI).	Median (range) baseline number of correct answers on the scenarios before the intervention was 9 (3–11) and increase to 11 (9–12) with the use of the app (p<0.001). The median (range) demonstration score was 6 (5–6) for the video training module group and 4.5 (3–6) for the label group (p<0.001).	To support and improve traditional methods for anaphylaxis training, a mobile health decision support technology is useful and feasible.
Soller, 2018 [34]	Before and after study.	Canada (n=353 OFC).	Parents and children (18 years) on an in-hospital OFC. Food allergy.	Training of symptoms and signs of anaphylaxis and EAI use during actual episodes of anaphylaxis.	Pre- and post-challenge questionnaire.	4 domains assessed: conﬁdence in the ability to recognize a severe allergic reaction, conﬁdence in EAI administration, perceived technical knowledge of EAI technique, and perceived skill in EAI use.	Recognition of anaphylaxis: Mean: pre-challenge: 3.58 (95%CI, 4.49-3.67) - post-challenge 3.96 (95%CI, 3.74, 4.18). Administration: pre-challenge: 3.25 (95%CI,3.14-3,36) - post-challenge 4.23 (95%CI, 4.01, 4.45). Knowledge: pre-challenge: 3.61 (95%CI, 3.50-3.72) - post-challenge 4.26 (95%CI, 4.04, 4.28). Skill: pre-challenge: 2.73 (95%CI, 2.61-2.85) - post-challenge 3.85 (95%CI, 3.63, 4.07).	It is possible to supervise the administration of EAIs during OFC to increased conﬁdence in the recognition of anaphylaxis and knowledge of EAI use.
Alqurashi W. 2020 [22]	Observational study.	Canada (N=230).	Pediatric population <17 years of age. All types of allergies.	To validate the Kids’ CAP, to assess its impact on anaphylaxis recognition and treatment, and to determine its perceived usefulness.	No comparison.	Development phase: Readability. Understandability and Actionability (Patient Education Materials Assessment Tool for Printable Materials). Clinical phase: Health literacy (Newest Vital Sign); Quality of the written medical information, comprehensibility, design quality, and usability (Consumer Information Rating Form); Comprehension (Kids’ CAP Comprehension Assessment)	Development phase: The infographic scored an average GFI of 9 and an FRG score of 5; The understandability and actionability of the Kids’ CAP were deemed acceptable in the ﬁrst review with median scores of 88% (range 84%–92%) and 85% (range 71%–100%), respectively. Clinical phase: Of the 230 participants enrolled, 205 (89%) completed the follow-up interview. The written contents of the Kid's CAP were modified to match grade 7 readability level. The total mean score of the CIRF for comprehensibility was 23.1 (SD 2.4), and 25.1 (SD 2.3) for design quality. The mean comprehension score was 11.3 (SD 1.8) (reference range 0-12), with no significant difference between participants with and without previous experience with anaphylaxis, or high vs. low literacy level.	In designing action plans to prevent the effects of anaphylaxis, it is very useful to engage children and parents in the design and contents of plans.
Korematsu. 2022 [31]	Observational study.	Japan (n=597 institutions).	All public or private elementary schools, junior high schools, and high schools. Compulsory education schools: special-needs schools. Public and private kindergartens. certified childcare facilities, and day-care centers in the prefecture (1.118 institutions). All types of allergies.	Implementation of guidelines.	Online questionnaire after the implementation.	Detection of symptoms of children with an EAI was recommended.	Among the 48 children who had symptoms for which an EAI was recommended; 23 had symptoms based on the evaluation within 30 seconds (Look sick, difficult to breathe, decreased consciousness) and 25 had symptoms based on the evaluation within 5 minutes (systemic, respiratory, and digestive symptoms).	There is a need for appropriate response training to anaphylaxis in schools, kindergartens, childcare facilities, and nursery schools including those which do not provide school lunch services or do not have children diagnosed with FAs.
Efthymiou. 2021 [35]	Observational study.	Cyprus (n=11 schools).	Personnel of preschool facilities and schools. Food allergy.	Evaluation of allergy management competencies in primary schools with a questionnaire (42 questions).		Recognition of signs and symptoms of anaphylaxis. Training and preparedness.	Eight on eleven respondents stated that they know some of the signs of FA. They mentioned 7 types of signs and symptoms: wheals (4/11), itching (2/11), airway obstruction (2/11), wheezing (2/11), dyspnoea (1/11), abdominal pain (2/11) and oedema (1/11), but 3/11 could not recall any symptom. The personnel had received training relevant to allergies and allergic symptoms and were prepared to manage an allergic reaction in a child, in only 2/11 schools where seminars had been provided twice in the preceding 3 years, by an allergist and a dietitian.	The most recognized symptoms were wheals, itching, airway obstruction, and wheezing and there is a need for protocol and training of primary school personnel to improve recognition and management of FA.
Esenboga. 2020 [24]	Observational study.	Turkey (N=190).	Patients aged 1 to 18 years who were prescribed EAIs for any reason. All types of allergies.	To determine attitudes and knowledge levels of patients/parents regarding the use of EAIs with face-to-face interviews (95%) and a survey for parents.	Face-to-face interview or by telephone (95%). Parents completed a survey.	Experience of anaphylaxis. Use, carriage, and storage of EAI.	Forty-four parents’ experiences anaphylaxis and indicated in order as symptoms: Itching, urticaria, angioedema (≈37%); Breathing difficulty (≈34%); Tightening in the throat or chest (≈18%); Dizziness, fatigue, near fainting (≈4%); Repeated vomiting (≈2%); Resistant severe cough (≈13%).	Patients’ and parents’ concerns and fears should be taken into consideration and necessary support should be provided after the prescription of EAI. Repeat training sessions and psychological support are useful.
Glassberg B. 2021 [27]	Observational study.	United States of America. (n=200).	Caregivers of pediatric patients with food allergies. FA.	To understand the factors associated with the underuse of EAI by caregivers of pediatric patients.	Survey.	EAI use. Reasons for not administering EAI.	164 surveys were completed; 118 (72%) of lifetime most severe reactions warranted EAI use, but the EAI was used in only 45 (38.1%). Reasons caregivers indicated for not administering the EAI: reactions did not seem severe enough; it was the patient's first allergic reaction; use of other medication; and fear of using EA.	Multiple factors contribute to the underuse of EAI in the treatment of severe allergic reactions. This study highlights the need for ongoing education for caregivers and pediatric patients with FA, based on recognition of the signs and symptoms of anaphylaxis to alleviate the fear of EAI use.

As we did not identify any studies that directly addressed our research question, we selected articles that indirectly related to our search. We selected 10 studies (Table 4), two experimental studies [20,21], and eight observational studies [23,25,26,28,30,32-34] about the impact of an educational intervention in recognition, management of anaphylaxis and epinephrine auto-injector (EAI) use. Two retrospective studies described the effect of the implementation of an action plan or new protocol on knowledge of anaphylaxis recognition and treatment [22,31]. Four studies assessed knowledge about the recognition and management of anaphylaxis in specific populations [24,27,29,35] and on review identified a lack of knowledge to enable recognition of anaphylaxis as a factor associated with the underuse of EAI [27].

**Table 4:** Classification of studies according to the scope of intervention

Scope of intervention	Number of studies	Type of studies
Effect of educational intervention	10	2 experimental studies [20,21], 8 observational studies [23,25,26,28,30,32-34]
Effect of the implementation of an action plan or protocol	2	2 retrospective studies [22,31]
Assessment of knowledge	4	4 descriptive studies [24,27,29,35]
Assessment of knowledge as a factor associated with underuse of epinephrine	1	1 descriptive study [27]

Educational interventions

One RCT [20], one controlled before-and-after study [21], and five observational studies [23,25,28,32,33] assessed the effect of a training session on knowledge of signs and symptoms of anaphylaxis and anaphylaxis management. Three other observational studies assessed the effect of a different form of educational intervention [26,30,34].

In the RCT, Brockow et al. measured the difference in knowledge about anaphylaxis (questionnaire) and the competence level in the management of a simulated anaphylactic reaction in 193 participants (95 caregivers and 98 patients) before and three months after a course with two three-hour schooling modules [20]. In comparison with the control group (CG) that did not follow a training module, the intervention group (IG) was shown to have a significant improvement in knowledge for both caregivers (IG 3.2/13.2 improvements/baseline vs CG 0.7/12.6; P<0.001) and patients (IG 3.9/10.8 vs 1.3/12.6; P<0.001).

In a controlled before and after study, Canon et al. evaluated the role of a 1-hour educational session on food allergies and measured its efficacy for improving knowledge in 375 teachers from six private schools randomly assigned into an intervention group (n = 4 schools) and a control group (n = 2 schools) [21]. The post-test intervention group had a knowledge score 19.58% points higher than the control group (95%CI, 16.62-22.53; P<0.001), with no differences in pretest scores. Pretest knowledge score values were higher in teachers who had a graduate school education (9.5%; 95%CI, 0.45-18.52; p=0.04) and a college education (10.4%; 95%CI, 0.70-20.10; P=0.036) versus those who did not complete college. 

Five observational studies with a total of 974 participants assessed knowledge in recognition of signs and symptoms of anaphylaxis and anaphylaxis management before and after a training session without a comparison group [23,25,28,32,33]. In 53 unlicensed high school assistive personnel, knowledge assessment in recognition of signs and symptoms of anaphylaxis and perceived self-efficacy in EAI administration revealed a significant improvement following the intervention (p<0.001) [32]. The same result was found in another study in a population of 53 participants (80% teachers, 20% canteen staff), in which 39.6% recognized anaphylaxis in the pre-training questionnaire and 81% in the post-training questionnaire (p<0.001) [25]. Likewise, the proportion of school staff who believed they knew when to use an EAI increased from 19% to 100% (p<0.001). In another study, Polloni et al. reported a baseline low self-efficacy in anaphylaxis management, especially in recognizing anaphylaxis symptoms and administering proper drugs [33]. All scores concerning self-efficacy in anaphylaxis management of 592 teachers and school caretakers improved after the course with a statistically significant difference in recognition of anaphylaxis symptoms as measured by the School Personnel Self-Efficacy-Food Allergy and Anaphylaxis Questionnaire (S.PER.SE-FAAQ) (I quartile=0.0; median=1.0; mean 1.1; III quartile=2.0; p<0.05).

Improvement was also reported by Gonzales-Mancebo et al. in a study of 191 summer camp attendees (24% of teachers, 13% of cooks, 51% of cafeteria monitors, and 12% of camp leaders or others) where they found a significant difference in the mean score (MS) before and after the training for questions about recognition of anaphylaxis symptoms (before training: MS=3.64; SD=1.14; After training: MS=4.56; SD=0.76; p<0.05) and administration of drugs to a student with severe reaction (before training: MS=3.08; SD=1.41; After training MS=4.51, SD=0.84; p<0.05) [28]. An improvement following an educational intervention was also found by Dumeier et al. in a population of 75 preschool teachers [23]. In this study, the knowledge of clinical signs increased significantly after the training session for three symptoms: anal and urinary incontinence (23% at baseline, 81% directly after the education session; 23% at 4 to 12 weeks after the education session, p<0.025); dip in blood pressure and dizziness (65% at baseline, 91% directly after the education session, 87% at 4 to 12 weeks after the education session, p<0.025) and for nausea and vomiting (59% baseline, 89% directly after the education session, 80% at 4 to 12 weeks after the education session, p<0.025). However, no difference was found for two other symptoms, shortness of breath and wheezing, and for swelling of skin and mucosa, which was known before the training course by 56% (shortness of breath/wheezing) and 73% (swelling of skin/mucosa) of participants. A statistically significant difference was also identified for knowledge of all five symptoms correctly related (9% baseline, 60% directly after the education session, 31% at 4 to 12 weeks after the education session; p<0.025).

Three observational before and after studies with a total of 517 participants assessed the effect of new forms of educational interventions [26,30,34]. The efficacy of a peer-to-peer educational video in increasing food allergy knowledge was assessed in a population of children and adolescents (198 elementary, 156 middle, and 203 high school students) [30]. Knowledge scores of common food allergy (FA) symptoms increased significantly among students after viewing the videos (elementary school: 61.5% at the pre-test vs. 85.9% at the post-test, p<0.001; middle school: 46.3% vs. 70.1%, p<0.001; high school: 66.1% vs. 85.2%, p<0.001) and more than 60% of students reported that they learned something new about FA. The use of a smartphone health app for the management of patients with potentially life-threatening FA was studied by Gallagher et al. [26]. Twenty-two adolescents (13 to 19 years) were asked to solve 12 clinical case quizzes with and without the use of the app. The median (range) correct score out of 12 for the baseline testing was 9 (3-11). After the utilization of the app’s decision support function, scenario testing median scores increased to 11 (9-12), p<0.001. In the last educational study, Soller et al. assessed the efficacy of children and parents coached by clinicians during an oral food challenge (OFC) [34]. In this study, parents and their children (<18 years old) were invited to administer the EAI under the supervision of a nurse/allergist if anaphylaxis occurred during the OFC and to complete a questionnaire before and after the intervention. A total of 353 OFCs were performed, with 5.6% developing an anaphylactic reaction. Epinephrine was used in 15.0% of the anaphylactic reactions and was administered by the parent (69.8%), child (26.4%), or practitioner (3.8%). Pre-challenge and post-challenge mean confidence scores (MS) were statistically significant for recognition of anaphylaxis (pre-challenge MS=3.58; 95%CI, 3.49-3.67; post-challenge MS=3.96; 95%CI, 3.74-4.18), and for knowledge of EAI use (pre-challenge MS=3.61; 95%CI, 3.50-3.72; post-challenge MS=4.26; 95%CI, 4.04-4.28).

Action plan and protocol

Two observational studies assessed the effect of the implementation of an action plan or new protocols [22,31]. In the first study, 205 participants (31 children and 174 parents of children) were involved in appraising the design and written contents of the Canadian Anaphylaxis Action Plan for Kids [22]. The overall comprehension and knowledge of anaphylaxis management including recognition of anaphylaxis were objectively assessed through four hypothetical scenarios after the implementation of the action plan. Out of a maximum score of 12 for the Comprehension Assessment Questionnaire, the mean knowledge score was 11.3 (SD=1.8; range, 0-12). There was no significant difference between parents versus children, participants with versus without previous anaphylaxis experience, or high versus low literacy level. However, parental education level was the only factor associated with a statistically significant difference in knowledge scores between study participants; the mean knowledge score for parents with a college education or higher was 11.4±1.7 (SD) compared with the mean knowledge score for parents without college education (10.4±1.2; Mean Difference=1; 95%CI, 0.5-0.6, p<0.001). 

In the second study, a fact-finding survey aimed at determining if appropriate responses to anaphylaxis onset were implemented in schools, kindergartens, childcare facilities, and nursery schools in a Japanese town [31]. This survey was conducted four years after the issuance of new guidelines focused on the signs and symptoms of anaphylaxis and their time of onset. Five hundred and ninety-seven institutions responded to the questionnaire, showing the underutilization of EAIs (three uses of EAIs on 48 anaphylactic reactions) secondary to the absence of prescriptions and the insufficiency of training on anaphylaxis management.

Knowledge

Four observational studies assessed knowledge about signs and symptoms of anaphylaxis in a population of patients and parents [24,27], schoolteachers [35], and nannies [29]. In a study conducted in a population of caregivers of pediatric patients with food allergies [27], 164 questionnaires were completed. All but one of the caregivers reported previously receiving education about EAI, and all participants reported at least one past food-related allergic reaction in their child. The most typically reported symptoms at the time of most severe reactions are reported in Table 5. In the second study, 190 patients (1 to 18 years) who were prescribed EAIs were invited with their parents to a face-to-face interview and to complete a survey to evaluate their attitudes and knowledge levels to provide standardized and better education [24]. One-fourth of EAI-prescribed patients experienced anaphylaxis requiring the use of an EAI within the previous ﬁve years and 30% of the patients used an EAI. Regarding signs and symptoms, 44 patients who experienced anaphylaxis reported the following signs and symptoms during anaphylaxis: itching, urticaria, angioedema (≈37%), breathing difficulty (≈34%), tightening in the throat or chest (≈18%), dizziness, fatigue, near fainting (≈4%), repeated vomiting (≈2%), resistant severe cough (≈13%).

**Table 5:** Typical symptoms reported with severe food allergy reaction ^a ^Symptoms include the following: dizziness, unexpected urination, swelling of extremities, a feeling of dread, confusion, sleepiness, change in color, runny nose, watery/glossy eyes, bad taste in the mouth, itching of ears, swelling of face, sneezing, raspy voice/change in voice, runny nose, and bloody nose. Glasberg et al. [27]

Symptoms at the time of most severe reaction	n	%
Hives	139	84.8
Pruritus	82	50.0
Vomiting	68	41.5
Swelling of lips	62	37.8
Itching in throat	61	37.2
Cough	55	33.5
Swelling of eye	52	31.7
Trouble breathing	51	31.1
Wheezing	46	28.0
Pain in stomach	31	18.9
Change in behavior	27	16.5
Swelling of tongue	16	9.8
Others^a^	14	8.5
Chest tightening	13	7.9
Diarrhea	10	6.1
Low blood pressure	4	2.4
Loss of consciousness	3	1.8

In another study, Efthymiou et al. examined the knowledge and beliefs of schoolteachers about food allergy in 11 primary schools in Cyprus [35]. The personnel had received training relevant to allergies in only two of 11 schools (18.2%). Regarding recognition of the signs and symptoms, eight schools out of 11 that responded to a questionnaire stated that they had identified some of the signs of food allergy. They described seven different signs and symptoms: wheals (4/11), itching (2/11), airway obstruction (2/11), wheezing (2/11), dyspnea (1/11), abdominal pain (2/11), and oedema (1/11), but 3/11 could not recall any symptom. The last study identified gaps in knowledge of a nanny’s population [29]. Ninety-nine percent of nannies interviewed (n=153) recognized food allergies as a potentially fatal event. Considering signs of food allergies, 51% were comfortable with recognizing a food allergy emergency, and 49% for treating this allergy emergently. Most nannies (66%) desired additional information about recognizing and treating food allergies. 

Factors associated with the underuse of epinephrine auto-injectors

One review and meta-analysis identified in our search strategy investigated potential barriers to epinephrine use [19]. In a narrative review of 23 studies, Miles et al. identified potential barriers to epinephrine used at school. Low availability of epinephrine, insufficient education and training in anaphylaxis recognition and management, and insufficient policies or protocol were the main barriers to epinephrine use in the school setting. For insufficient education and training on anaphylaxis management, studies included in this review report that about half of respondents, composed of school staff (i.e., nurses, teachers, and principals), knew to use epinephrine as first-line treatment to manage anaphylaxis; however, a much lower proportion (5%-35%) knew how or when to use an EAI. Four studies found low self-efficacy in anaphylaxis management among school personnel as well as a lack of training [19].

The same search from September 2022 was rerun on April 4, 2023 to identify any additional studies since our last search and before publishing this scoping review. One RCT was identified which described the effect of a mobile web-based food allergy and anaphylaxis management educational program for the recognition and management of anaphylaxis [36]. The findings of this study will be included in a planned upcoming systematic review of educational interventions for the recognition and care of anaphylaxis.

## Discussion

Why this topic was reviewed

The purpose of this scoping review was to examine the literature since the 2010 ILCOR CoSTR [16] to establish whether there was new evidence in published and gray literature regarding first aid providers’ ability to recognize anaphylaxis.

Summary of our findings

We have not identified any new data in the published and gray literature to suggest that the presence or absence of any specific symptom may improve the accuracy of recognizing anaphylaxis by the first-aid provider. We have extended our eligibility criteria and selected articles to include individuals who may be first aid providers as patients, parents, teachers, school staff, and nannies or babysitters. We have selected articles that do not directly assess the signs or symptoms of anaphylaxis but rather how to assess and improve the level of knowledge of participants in the recognition and management of anaphylaxis with the aim that more patients with anaphylaxis can receive epinephrine. Two experimental studies and eight observational studies show an increase of knowledge in the recognition of anaphylaxis in a population of patients, parents, school staff, or caregivers after an educational intervention as a theoretical and practical course, viewing videos, health app use, or coaching with clinicians [20,21,23,25,26,28,30,32-34]. The use and the effect of an action plan and the dissemination of specific recommendations for patients and in school communities are described in two studies with no demonstrated level of effectiveness [22,31]. The need for learning and training of the population seems indeed important and the absence of training in recognition of anaphylaxis is identified as a factor of underuse of epinephrine [19]. Even if the initial level of knowledge in recognition of anaphylaxis before an educational intervention is low and increased after an educational intervention, the level of knowledge, recognition, and management of anaphylaxis remains low a few months or a year later [24,27,29,35].

Implications of our work

Epinephrine is a potentially life-saving intervention for anaphylaxis. The ability of a first aid provider to recognize anaphylaxis is a critical step prior to administering epinephrine. The studies identified in this scoping review are encouraging, with several surveys reporting improvement in the ability to recognize anaphylaxis immediately following individual or community-level educational engagements. New local policies and implementation of action plans or protocols about recognition and management of anaphylaxis provided for patients or for some settings deserve further studies to show their effectiveness in the short and long term. The content and form of the action plan or protocols should also be analyzed as they could affect its efficiency.

Compare and contrast with previous literature

Previous literature has identified different factors contributing to the underuse of epinephrine in anaphylaxis [6,37]. Recognition of anaphylaxis is one of these identified factors which facilitates the administration of epinephrine when it is available even though the evidence is limited. Recognition of anaphylaxis is not alone. The high cost of epinephrine, lack of epinephrine availability among patients or in some settings such as schools, lack of epinephrine use even when available, and incorrect technique to administrate EAIs are also identified as barriers to the use of epinephrine by patients and first aid providers when they need it.

How do we advance the science, where do we go from here?

The results of this scoping review led to two considerations. First, the previous treatment recommendation of the 2010 ILCOR CoSTR continues to be supported by the limited evidence identified: “First aid providers should not be expected to recognize the signs and symptoms of anaphylaxis without repeated episodes of training and encounters with victims of anaphylaxis” [16]. Second, it appears that there is sufficient evidence to consider for a future systematic review to compare educational approaches to teaching and training lay providers to improve their ability to recognize signs and symptoms and to manage care for a person with anaphylaxis.

Limitations

Our scoping review has several limitations. Our only outcome in the research question is anaphylaxis recognition. No other outcomes are examined but we have not found any articles with other outcomes for this topic, including clinical outcomes. All articles selected were conducted in high-income countries and the results of our research cannot be extrapolated to low- or middle-income countries. The definition of anaphylaxis varied between studies. Skin involvement was not always included in the diagnostic criteria and some studies did not include the definition of anaphylaxis. The included studies assessed the effect of an educational intervention using a survey, or a test completed before and immediately after the intervention. None of the studies show their effectiveness using a real-life scenario or demonstrate clinical outcomes to show persistent improvements that may be associated with the educational intervention. Most studies report results for signs and symptoms of anaphylaxis and do not indicate if they include the medical history of patients in the set of elements for the recognition of anaphylaxis. We were, therefore, unable to examine the importance of patients' medical history as a criterion for the recognition of anaphylaxis independently.

## Conclusions

One of the most concerning aspects of anaphylaxis is the general global rise in the number of cases. Epinephrine remains the potentially life-saving gold standard treatment for a person with anaphylaxis. The inability of a first aid provider to recognize anaphylaxis is an identified major barrier to the use of epinephrine auto-injectors. The studies identified in this scoping review are encouraging, with several surveys reporting improvement in the ability to recognize anaphylaxis immediately following individual- or community-level educational engagements. New local policies and implementation of action plans or protocols about recognition and management of anaphylaxis provided for patients or for some settings deserve further study to show their effectiveness in the short and long term. Future studies should examine how educational interventions, local policies, and specific action plans can improve the recognition and management of anaphylaxis by first aid providers. Studies are also needed to evaluate clinical outcomes following educational interventions to improve the recognition and management of anaphylaxis.

While this scoping review did not identify studies that directly address the ability of first aid providers to identify anaphylaxis, most of the included studies focus on the education of lay responders in the recognition and management of anaphylaxis, with outcome measures of knowledge and confidence to use an EAI. A future systematic review is planned to compare educational modalities on outcomes of recognition and management of anaphylaxis.

## Appendices

Medline® with PubMed 2022 search strategies

Results from Medline® with PubMed search strategy from October 22, 2019 to September, 19, 2022 for the 2022 ILCOR First Aid Task Force scoping review on the ability of first aid providers to recognize anaphylaxis are presented in Table 6.

**Table 6:** Medline® with PubMed 2022 search strategies from 22 October 2019 to 19 September 2022 Date searched: September 19, 2022

#	Searches	Number of Articles
1	(recogni*[Title/Abstract] OR knowledge*[Title/Abstract] OR skill*[Title/Abstract] OR educat*[Title/Abstract] OR information*[Title/Abstract] OR train*[Title/Abstract]) AND (anaphyla*[Title/Abstract] OR epinephrin*[Title/Abstract] OR adrenalin*[Title/Abstract] OR epi-pen*[Title/Abstract] OR epipen*[Title/Abstract])	1162
2	((underus*[Title/Abstract] OR under-us*[Title/Abstract] OR underutili*[Title/Abstract] OR under-utili*[Title/Abstract]) AND (anaphyla*[Title/Abstract] OR epinephrin*[Title/Abstract] OR adrenalin*[Title/Abstract] OR epi-pen*[Title/Abstract] OR epipen*[Title/Abstract]))	21
3	((comfort*[Title/Abstract] OR discomfort*[Title/Abstract] OR dis-comfort*[Title/Abstract] OR uncomfortable[Title/Abstract] OR confiden*[Title/Abstract] OR empower*[Title/Abstract]) AND (anaphyla*[Title/Abstract] OR epinephrin*[Title/Abstract] OR adrenalin*[Title/Abstract] OR epi-pen*[Title/Abstract] OR epipen*[Title/Abstract]))	344
4	(manage*[Title/Abstract] AND anaphyla*[Title/Abstract])	700
5	#1 or #2 or #3 or #4	1835
6	"Patient Education as Topic"[Mesh]	3351
7	"Self Administration"[Mesh]	844
8	"Self-Management"[Mesh]	2628
9	(layperson*[Title/Abstract] OR lay-person*[Title/Abstract] OR laypeople*[Title/Abstract] OR lay-people*[Title/Abstract] OR nonprofessional*[Title/Abstract] OR non-professional*[Title/Abstract])	1706
10	(parent[Title/Abstract] OR parents[Title/Abstract] OR parental[Title/Abstract] OR communit*[Title/Abstract] OR teacher*[Title/Abstract] OR caregiver*[Title/Abstract] OR care-giver*[Title/Abstract] OR personnel*[Title/Abstract] OR school*[Title/Abstract] OR child care worker*[Title/Abstract] OR childcare worker*[Title/Abstract] OR aide*[Title/Abstract])	323405
11	(patient*[Title/Abstract] AND (educat*[Title/Abstract] OR train*[Title/Abstract] OR manage*[Title/Abstract] OR instruct*[Title/Abstract] OR confiden*[Title/Abstract] OR complian*[Title/Abstract] OR adheren*[Title/Abstract]))	378510
12	self-manage*[Title/Abstract]	7869
13	"First Aid"[Mesh]	349
14	"Emergency Medical Technicians"[Mesh]	281
15	first aid*[Title/Abstract] OR first respon*[Title/Abstract] OR EMT[Title/Abstract] OR emergency medical technician*[Title/Abstract] OR paramedic*[Title/Abstract] OR para-medic*[Title/Abstract] OR ambulance*[Title/Abstract]	17145
16	#6 OR #7 OR #8 OR #9 OR #10 OR #11 OR #12 OR #13 OR #14 OR #15	686475
17	#5 AND #16	955
18	("Animals"[Mesh] NOT ("Animals"[Mesh] AND "Humans"[Mesh]))	345367
19	#17 NOT #18	953
20	comment[Publication Type] OR editorial[Publication Type] OR news[Publication Type] OR newspaper article[Publication Type]	231609
21	#19 NOT #20	952

Embase 2022 search strategies

Results from Embase search strategy with Embase interface from October 22, 2019 to September, 19, 2022 for the 2022 ILCOR First Aid Task Force scoping review on the ability of first aid providers to recognize anaphylaxis are presented in Table 7.

**Table 7:** Embase 2022 search strategies from 22 October 2019 to 19 September 2022 Date searched: September 19, 2022

#	Searches	Number of Articles
1	(recogni*:ti,ab,kw OR knowledge*:ti,ab,kw OR skill*:ti,ab,kw OR educat*:ti,ab,kw OR information*:ti,ab,kw OR train*:ti,ab,kw) AND (anaphyla*:ti,ab,kw OR epinephrin*:ti,ab,kw OR adrenalin*:ti,ab,kw OR 'epi pen*':ti,ab,kw OR epipen*:ti,ab,kw)	2185
2	(underus*:ti,ab,kw OR 'under us*':ti,ab,kw OR underutili*:ti,ab,kw OR 'under utili*':ti,ab,kw) AND (anaphyla*:ti,ab,kw OR epinephrin*:ti,ab,kw OR adrenalin*:ti,ab,kw OR 'epi pen*':ti,ab,kw OR epipen*:ti,ab,kw)	38
3	(comfort*:ti,ab,kw OR discomfort*:ti,ab,kw OR 'dis comfort*':ti,ab,kw OR uncomfortable:ti,ab,kw OR confiden*:ti,ab,kw OR empower*:ti,ab,kw) AND (anaphyla*:ti,ab,kw OR epinephrin*:ti,ab,kw OR adrenalin*:ti,ab,kw OR 'epi pen*':ti,ab,kw OR epipen*:ti,ab,kw)	602
4	manage*:ti,ab,kw AND anaphyla*:ti,ab,kw	1265
5	#1 OR #2 OR #3 OR #4	3307
6	'patient education'/exp	11503
7	'drug self administration'/exp	1716
8	'self medication'/exp	1439
9	(layperson*:ti,ab,kw OR 'lay person*':ti,ab,kw OR laypeople*:ti,ab,kw OR 'lay people*':ti,ab,kw OR nonprofessional*:ti,ab,kw OR 'non professional*':ti,ab,kw)	1407
10	(parent:ti,ab,kw OR parents:ti,ab,kw OR parental:ti,ab,kw OR communit*:ti,ab,kw OR teacher*:ti,ab,kw OR caregiver*:ti,ab,kw OR 'care giver*':ti,ab,kw OR personnel*:ti,ab,kw OR school*:ti,ab,kw OR 'child care worker*':ti,ab,kw OR 'childcare worker':ti,ab,kw OR 'childcare workers':ti,ab,kw OR aide*:ti,ab,kw)	302975
11	patient*:ti,ab,kw AND (educat*:ti,ab,kw OR train*:ti,ab,kw OR manage*:ti,ab,kw OR instruct*:ti,ab,kw OR confiden*:ti,ab,kw OR complian*:ti,ab,kw OR adheren*:ti,ab,kw)	536453
12	'self manage*':ti,ab,kw	7863
13	'layperson'/exp	736
14	'first aid'/exp	935
15	'rescue personnel'/exp	963
16	('first aid*':ti,ab,kw OR 'first respon*':ti,ab,kw OR emt:ti,ab,kw OR 'emergency medical technician*':ti,ab,kw OR paramedic*:ti,ab,kw OR 'para medic*':ti,ab,kw OR ambulance*:ti,ab,kw)	20530
17	#6 OR #7 OR #8 OR #9 OR #10 OR #11 OR #12 OR #13 OR #14 OR #15 OR #16	825625
18	#5 AND #17	1880
19	#18 NOT ([conference abstract]/lim OR [editorial]/lim OR [erratum]/lim OR [letter]/lim OR [note]/lim)	954
20	#19 NOT ([animal cell]/lim OR [animal experiment]/lim OR [animal model]/lim OR [animal tissue]/lim)	946

Cochrane 2022 search strategies

Results from Cochrane with Central interface search strategy from October 22, 2019 to September, 19, 2022 for the 2022 ILCOR First Aid Task Force scoping review on the ability of first aid providers to recognize anaphylaxis are presented in Table 8.

**Table 8:** Cochrane 2022 from 22 October 2019 to 19 September 2022 Date searched: September 19, 2022

#	Searches	Number of Articles
1	((recogni* or knowledge* or skill* or educat* or information* or train*) AND (anaphyla* or epinephrin* or adrenalin* or epi-pen* or epipen*)):ti,ab,kw	361
2	((underus* or under-us* or underutili* or under-utili*) and (anaphyla* or epinephrin* or adrenalin* or epi-pen* or epipen*)):ti,ab,kw	3
3	((comfort* or discomfort* or dis-comfort* or uncomfortable or confiden* or empower*) and (anaphyla* or epinephrin* or adrenalin* or epi-pen* or epipen*)):ti,ab,kw	260
4	(manage* AND anaphyla*):ti,ab,kw	93
5	#1 OR #2 OR #3 OR #4	597
6	MeSH descriptor: [Patient Education as Topic] explode all trees	9294
7	MeSH descriptor: [Self Administration] explode all trees	787
8	MeSH descriptor: [Self-Management] explode all trees	714
9	(layperson* OR lay-person* OR laypeople* OR lay-people* OR nonprofessional* OR non-professional*):ti,ab,kw	331
10	(parent OR parents OR parental OR communit* OR teacher* OR caregiver* OR care-giver* OR personnel* OR school* OR 'child care worker*' OR 'childcare worker*' OR aide*):ti,ab,kw	46277
11	(patient* AND (educat* OR train* OR manage* OR instruct* OR confiden* OR complian* OR adheren*)):ti,ab,kw	82181
12	(self-manage*):ti,ab,kw	3404
13	MeSH descriptor: [First Aid] explode all trees	99
14	MeSH descriptor: [Emergency Medical Technicians] explode all trees	181
15	(first aid* OR first respon* OR EMT OR emergency medical technician* OR paramedic* OR para-medic* OR ambulance*):ti,ab,kw	20561
16	#6 OR #7 OR #8 OR #9 OR #10 OR #11 OR #12 OR #13 OR #14 OR #15	140925
17	#5 AND #16	409

Gray literature search strategies executed in Google from 2019 to 2022

Results from the gray literature search strategy executed in Google from September 19, 2019 to September 19, 2022 for the 2022 ILCOR First Aid Task Force scoping review on the ability of first aid providers to recognize anaphylaxis are presented in Table 9.

**Table 9:** Gray literature search strategies executed in Google from 2019 to 2022 Date searched: September 30, 2022

#	Searches	Results (2019-2020)	Results screened	New potentially relevant records	Total records	Included after review
1	anaphylaxis AND recognition	~ 12 300	100	18	3	1
2	anaphylaxis AND recognize	~ 16 200	100	12	3	1
3	anaphylaxis AND identification	~ 17 600	100	8	0	0
4	anaphylaxis AND identify	~ 17 400	100	3	0	0
